# Conference Review: ACHS FUTURES 2020. 5th Association of Critical Heritage Studies Biennial Conference. University College London, 26th August—30th August 2020

**DOI:** 10.1186/s43238-020-00018-2

**Published:** 2020-12-20

**Authors:** Plácido González Martínez

**Affiliations:** College of Architecture and Urban Planning, Tongji University, 1239 Siping Road, Yangpu District, Shanghai, 200092 China

The fifth biennial Conference of the Association of Critical Heritage Studies (ACHS), entitled ACHS 2020: FUTURES, took place in London from August 26 thru August 30 2020. As part of the regular scientific meeting programs of the ACHS, this can be deemed as the most important event in the field of critical heritage studies, whose significance has been unexpectedly affected by the current pandemic situation.

The conference was chaired by Professor Rodney Harrison of the UCL Institute of Archaeology in association with the Arts and Humanities Research Council (AHRC) Heritage Priority Area. Being Harrison’s UCL group one of the most active in the field of critical heritage studies, there were great expectations about the Conference, understood as an invitation to broaden their recent project ‘Heritage: Futures’ to an international, massive debate. Not coincidentally, one month prior to the Conference, UCL published the outcomes of their research programme (Harrison et al. 2020), in a timely manner to add to the discussions for the Conference.

Adding to this, it is necessary to highlight how despite the original plans to celebrate the Conference as a face-to face event, the course of the COVID-19 epidemic raised the question within the organizers during the first months of 2020 about its future. According to Rodney Harrison, among the alternatives considered were its cancellation; its postponement; or its change of format. This led to the decision taken in April 2020 to shift to a fully virtual conference. This decision, which initially led to a situation of uncertainty for its complex technical implications, finally showed as the most appropriate, and will definitely determine the ways these events will be planned in the future.

Considering these two exceptional issues, the review of the Conference needs thus to point at them separately. The first one is a conceptual one; and it is to reflect to what extent the contents of the Conference have answered to the aims of the call for papers and the scope of critical heritage studies that gather international scholars in the ACHS. The second one is a formal one; and it is to evaluate to what extent the change of format of the Conference has contributed to the aims of the ACHS as an organization and to the definition of new approaches for scientific meetings of the ACHS in the future.

## Conference theme: futures

The Conference aimed to provide a critical perspective beyond a frequently stated aim of heritage, which is its capacity to influence the life of future generations. According to the call, the debate was posed differently, as an invitation to think how the idea of ‘futures’ determines the ways heritage is valued, documented, conserved and promoted. This engages with current open discussions like sustainable development, social justice and gender equality, among others: situations of conflict in contemporary societies, triggered by diverging visions about the future, that determine our current appreciation of the past.

By means of stirring these discussions, the Conference also aimed to pose questions about the future of critical heritage studies: questions, whose answers may come from an overview of the themes and sub-themes of previous four Conferences of the ACHS. For instance, issues of power have represented a constant concern since they were explicitly formulated in the first Conference in Gothenburg 2012; whereas discussions on intangible cultural heritage have traditionally offered an exceptionally contested realm. This is no less than the conservation of the built environment, also a continued conceptual thread which in the last years has been matched by discussions on the natural environment.

The London Conference has also evidenced new advancements from the previous 2018 Hangzhou Conference. Needless to say, that as a Conference for international scholars engaged in cultural, social and political discussions, they have been symptomatic of recent major turns in international policy, particularly from the US. Whereas the main topic in Hangzhou, ‘Borders’, evidenced the global consequences of the first divisive decisions of the Trump administration (e.g. the construction of the border wall with Mexico and the overly xenophobic tone of new legislation on immigration), the general spirit of the London Conference has been greatly determined by the last, no less divisive latest legacy of Trump (e.g. the inflammatory response to the Black Lives Matter movement, and governmental inaction against the pandemic crisis).

Adding to this determinant fact for the future genealogy of critical heritage studies, this review aims to point out at how the discussion on the future itself was a most challenging question. An especially pertinent one, in a field that crosses different disciplines, in a wide variety of cultural and political contexts, witnessing the enhanced role of heritage in contemporary societies. The presentations grouped together across 14 sub-themes; many of them included in the scope of the UCL’s ‘Heritage: Futures’ project: ‘Arts and Creative Practice’, ‘Future Policies and Politics of Heritage’, ‘Environmental Change and the Anthropocene’, ‘Digital Futures in and for Heritage’, ‘Folklore and Intangible Cultural Heritages’, ‘Heritage and Foodways’, ‘Conflict Heritage and Conflicted Heritages’, ‘Urban Heritage Futures’, ‘Future Methods and Approaches to Critical Heritage Studies’, ‘Future Landscapes of Heritage’, ‘The Future Museum: Collections and Collecting’, ‘Heritage and Time’, ‘The Futures of Heritage’, ‘Mobilities and Migration’.

Due to their ambition and diversity, the definition of sub-themes was representative, and at the same time, answered to the high expectations that the members of the ACHS had for the Conference. In total, the 14 sub-themes incorporated 138 oral sessions, with over 1100 submissions accepted and over 800 presentations delivered. These numbers, which speak of the success of the call and the interest that critical heritage studies rise among the scholarship, were also corresponded by the quality of the presentations and the subsequent discussions.

An important statement of the orientation of the Conference was the selection of all-female Keynote Speakers: Karen Salt; Sharon MacDonald; Kavita Singh and Dolly Jorgensen. This can be interpreted as a gesture from the organizers to rectify the omission of gender balance among the keynotes in the last ACHS Conference in Hangzhou 2018. Lectures from the keynote Speakers also reflected on key issues of current critical heritage discussions.

Salt’s address pointed at the issues of race, colonialism and the heritage of refusal, echoing the intense debate sparked by George Floyd’s killing and the widespread reactions from the Black Lives Matter movement in the summer of 2020, first in the US and later extended through the Western hemisphere. A discussion that continues raising questions about power and the continuity of structural racism that haunt multiple authorized heritage discourses.

MacDonald’s keynote focused on the awareness about how heritage practices may constitute an important avenue to consolidate the breach between society and the environment, by means of legitimizing and celebrating a history of human exploitation of resources and the consequent separation between culture and nature. This approach, which derives from the UCL project ‘Heritage Futures’ of which MacDonald is a member, aims to provide theoretical and practical alternatives to make heritage a vehicle toward sustainability.

Singh delivered her lecture on the topic of belonging, pointing at the future challenges of heritage where the process of secularization that gave birth to the modern conception of heritage is currently being reversed. Such reversal leads to a new scenario that challenges the notion of Outstanding Universal Value itself, and many of the conventions supporting the heritage discourse in the last 50 years.

Jørgensen’s lecture elaborated on the theoretical notions developed in MacDonald’s lecture, with a view on the specific issues to be found in nature related to the frequently understated phenomenon of animal extinction. Ethical and technical considerations about how to deal with the remnants of vanishing animal species, particularly those from the recent modern eras, shed light on wider discussions about the engagement of the heritage sector with the ongoing extinction crisis.

The attractiveness of sessions and presentations made the election where to surf in the program difficult. Even if the presentations were made available several days in advance of the Conference, time limitations continued to apply for what has been as massive scientific event: with choices multiplied, attendees finally focused on themes by means of affinity. The interest of this reviewer focused on the Urban Heritage Futures sub-theme, as well as in presentations related to urban and architectural heritage conservation which were dispersed in other sub-themes.

To this extent, it is important to highlight how scholars in architecture and the built environment have developed conceptual and methodological approaches that advance from the backward position to which these disciplines were relegated by Waterton and Watson (2013) in the field of critical heritage studies. Presentations like Piazzoni’s (2020) showed how the study of the monumental city of Rome can be completely subverted by means of introducing the perspective of migration and the transnational connections of Bangladeshi street vendors; very much in the line of Wulff’s (2020) studies on Islamic communities in Southern Italy.

Even if dealing with ‘traditional’ built heritage matters, the variety of presentations in sessions like Pendlebury and Wang’s on adaptive reuse, pointed at important questions in force in current specialised literature (Wang and Wang 2018; Liu et al. 2019; Pendlebury and Wang 2020). Among them, issues of authenticity seemed to keep their relevance, as it still remains a valid and contested notion around which issues of power and narratives revolve. Referring to the main question of the Conference, ‘Futures’, most of the presentations reflected on the agency that heritage has to produce images of the future (Veldpaus and Fava 2020), instead of answering to the question of how the definition of the future may be determining heritage processes today. Also in the same theoretical line, the approaches to reconstruction in presentations like Avrami’s (2020) for the hidden stories of slavery in Monticello; or Ishizawa’s (2020) on the re-creation of the ancient capital of Rwanda, showed how authenticity is still deemed a powerful tool to achieve either purposes of justice and aims of developmentalism.

These remarks are just a sample of the 42 presentations related to the field that this reviewer could attend. Moreover, the discussions on urban heritage conservation showed a breach between official approaches such as UNESCO’s Historic Urban Landscape (HUL) (UNESCO 2011), and their critical interpretation. It was surprising, for example, to see how the HUL Recommendation was absent in most of the presentations and debates. An interesting omission, when we are approaching 10 years since the Recommendation was passed, and in an international situation where HUL is still deemed as a key for management and conservation, and intensively used by UNESCO as a valuable training tool: are the ‘futures’ that the Conference aimed to discuss about, so detached from the course of international organizations?

## Conference format: moving towards an online future?

It might be coincidentally, but the breakthrough that the sub-themes proposed at the theoretical level has been deeply conditioned to the epidemic situation and the digital format towards which the Conference had to evolve. Due to the Conference submission calendar none of the presentations had the chance to elaborate about the pandemic; nevertheless, the whole Conference was embedded in an exceptional situation and the comments on the epidemic stood at the forefront in many of the discussions.

Even if the organizers met many challenges and there were doubts about the celebration of the event itself, it is necessary to highlight how they overcame the difficulties and made this an opportunity to critically rethink the format of future conferences. This deserves a particular comment, because it affects the nature of these academic discussions beyond the diversity of its topics. Due to the change of format to virtual and the subsequent reduced registration fee, the Conference could be appropriated by a wider community of scholars, particularly those coming from less affluent contexts, who initially had discarded to travel to London, one of the most expensive cities in the World.

Thanks to a smart and well thought plan of transition to digital, the chosen format enabled for a better appreciation of the discussions, as the contents were uploaded and made available online, leaving time for their careful study and evaluation. As they need to be prepared, it is this reviewer’s guess that the degree of satisfaction of the presenters with their performance has grown. Also, the physical scenario of lecture halls, which frequently functions as a highly pervasive environment where hierarchies are highlighted and discussions are prevented rather than encouraged, gave way to a virtual space which allowed for a more direct, horizontal interaction.

It may also be argued that on the other hand, there were things that were lost, and the direct, immediate face to face contact that so frequently gives way to other opportunities for networking and interaction, was not possible, especially for non-digital natives. Also, it is necessary to remind how barriers for digital inclusiveness like national firewalled networks, or insufficient broadband internet connection, remain in force for many scholars. But it is this reviewer’s contention that the benefits have overpassed the difficulties, and that the digital model is here to stay.

It is now an open current discussion within the ACHS for the format to adopt for the coming two conferences in Santiago 2022 and Maryland 2024: most probably, an intermediate mode will be the optimal solution, as it could conjugate the benefits of interaction and inclusiveness. Hopefully, once in the times to come the Internet may become universally accessible, we will look back at the London Conference as the turning point where the critical heritage sphere could definitely spread beyond the richest, most affluent societies in the World. What the heritage futures will look like then will be part of another discussion.

## Data Availability

Not applicable.
